# The association between global cognitive function and walking capacity in individuals with broad ranges of cognitive and physical function: Are there sex differences?

**DOI:** 10.3389/fresc.2022.960437

**Published:** 2022-09-12

**Authors:** Elise Wiley, Kenneth S. Noguchi, Kevin Moncion, Natalie D’Isabella, Daria A. Shkredova, Hanna Fang, Julie Richardson, Joy C. MacDermid, Lynden Rodrigues, Marc Roig, Ada Tang

**Affiliations:** ^1^ School of Rehabilitation Science, McMaster University, Hamilton, ON, Canada; ^2^ Department of Physiology, Radboud Institute for Health Sciences, Radboud University Medical Center, Nijmegen, Netherlands; ^3^ Centre for Heart, Lung, and Vascular Health, School of Health and Exercise Science, University of British Columbia, Kelowna, BC, Canada; ^4^ School of Physical Therapy, Western University, London, ON, Canada; ^5^ Memory and Motor Rehabilitation Laboratory (MEMORY-LAB), Feil and Oberfeld Research Centre, Jewish Rehabilitation Hospital, Montreal Center for Interdisciplinary Research in Rehabilitation, Laval, QC, Canada; ^6^ School of Physical / Occupational Therapy, McGill University, Montreal, QC, Canada

**Keywords:** global cognitive function, walking capacity, functional health, sex diferences, aging

## Abstract

**Introduction:**

Cognitive function is known to be associated with physical function, where greater walking capacity has been shown to have moderate to strong correlations with global cognitive function and other various domains of cognition in older adults with and without chronic conditions. Biological sex may moderate the relationship between cognitive and physical function, but whether sex differences exist in this association has not been examined in an aging population. The purpose of this study was to examine the associations between global cognitive function (Montreal Cognitive Assessment; MoCA), walking capacity (6-Minute Walk Test distance; 6 MWT) and sex in an aging population with broad ranges of cognitive and physical function.

**Methods:**

Participants were assessed for global cognitive function (MoCA) and walking capacity (6 MWT). Multivariable regression analyses were performed to examine the interaction of sex in the association between MoCA and 6 MWT. First, we presented the unadjusted model (Model 1), then the model adjusted for age, history of stroke, and height (Model 2). To determine if there were sex-based differences in the association between global cognitive function and walking capacity, we included sex and an interaction term between sex*6 MWT distance in Models 3 and 4.

**Results:**

Twenty-three females and 36 males were included in the multivariable regression analyses, respectively. Our sample represented broad ranges of cognitive and physical function levels, where MoCA scores ranged from 13 to 30, and 6 MWT distances from 203 to 750 m. 6 MWT distance was associated with MoCA in models unadjusted (*R*^2 ^= 0.17; *F*(1,56) = 11.4; *p* < 0.01) and adjusted for age, stroke history, and height (*R*^2 ^= 0.20; *F*(4,53) = 3.2; *p* = 0.02). No interaction with sex was found, but a main effect of sex was observed (*R*^2 ^= 0.26; *F*(5,21) = 3.72; *p* = 0.03). When adjusting for age, height and history of stroke, males MoCA scores were 2.9 ± 1.3 less than the mean MoCA scores for females.

**Discussion:**

Our findings confirm the positive relationship between cognitive and physical function in older adults. Notably, we also observed superior performance in global cognition among females that was consistent across a broad spectrum of walking capacity.

## Introduction

Mild cognitive impairment is present in approximately 40% of older adults worldwide (1). It can impact performance of activities of daily living (2), and increase the risk of depression, apathy, irritability, lowered quality of life (3), and cardiovascular disease (4, 5). Cognitive function is known to be associated with physical function. Conversely, greater walking capacity [i.e., 6-Minute Walk Test (6 MWT) (6) distance] has been shown to have moderate to strong correlations with global cognitive function (7–14), memory (8, 9, 15), attention (9, 14, 16), verbal fluency (9, 14), and executive function (9, 14, 17) in older adults with (6–11, 15–17) and without (12–14) chronic conditions. With increasing age, declines in cognition (18) and walking capacity (19), as well as higher prevalence of chronic conditions such as stroke (20), often occur. Age-associated changes in physical function (21, 22) can compromise the ability for older adults to engage in physical activity (23), potentially contributing to further declines in cognitive and functional capacity.

Biological sex may moderate the relationship between cognitive and physical function, as it strongly influences aging-related processes, as well as the prevalence, diagnosis, severity, and outcomes of disease (24). It is unclear however whether the relationship between global cognitive function and walking capacity favours male or female sex. Levels of circulating brain derived neurotropic factor (BDNF), which plays an important role in promoting neuronal growth, brain plasticity and synaptic interactions (25), and regulates spinal density of mature neurons (26, 27), are higher in females (28, 29). Higher BDNF levels has also been attributed to the improvements observed in executive function in females following aerobic exercise interventions (30–32). Older females may also experience slower age-related declines in physical function such as aerobic fitness (33–38) where recent research has shown that females appear to be more efficient in oxygen extraction and uptake during walking compared to males (39).

Conversely, other studies have shown that fitness and physical function may be preserved to a greater extent in older males relative to females (40), attributed to declines in estrogen levels in females, reduced bone density and muscle mass, and greater central adiposity which contribute to reduced physical activity levels (41). However, when factors such as blood volume and oxygen carrying capacity are taken into account, aerobic fitness may not differ between males and females (42).

Given the evidence of sex differences in neurotrophic factors favouring older females and possible between-sex differences in physiology that contribute to physical function abilities, it may be that sex moderates the association between cognitive function and walking capacity. Thus, the objective of this study was to examine the moderating effect of sex on the associations between global cognitive function and walking capacity in an aging population with a range of cognitive and physical abilities.

## Methodology

### Study design

To include participants with a broad range of cognitive and physical function that is representative of an aging population, this secondary data analysis included community-dwelling middle aged and older adults with and without stroke. Data were pooled from an observational cohort study of individuals with and without stroke (*n* = 34 and 17, respectively) [Hamilton Integrated Research Ethics Board (HIREB) 13-348] and baseline data from a randomized controlled trial in middle aged and older adults with stroke (*n* = 11) [HIREB 4713; Centre de Recherche Interdisciplinaire en Réadaptation du Montréal Métropolitain (CRIR-1310-0218), clinical trials registry NCT03614585]. All participants provided written informed consent.

### Participants

Participants without stroke were community dwelling older adults, 50–80 years old and able to walk at least 10 meters independently. Participants with stroke were 40–80 years old, ≥6 months following their first-ever stroke, living in the community, and able to walk at least 10-meters independently. We included the broader age range for individuals with stroke to account for the known mobility and cognitive impairment in this population. Individuals with stroke were excluded if they had a stroke of non-cardiogenic origin or tumor, scored <2 on the Modified Rankin Scale, had any contraindications to exercise testing (43), or class C or D American Heart Association Risk Criteria. Individuals were excluded if they possessed other neurological or musculoskeletal comorbidities, pain worsened with exercise, or cognitive, communication, or behavioural issues that could limit their ability to provide consent or follow instructions. In both studies, participants first completed their assessment of global cognitive function, which was subsequently followed by the assessment of walking capacity within the same visit to the lab. All participants were asked to abstain from food or drink for 4 h, smoking, caffeine and alcohol for 12 h and exercise for 24 h before each visit.

### Assessments

Demographic information, including age, biological sex, and medical history were obtained in all participants. Stroke-related information were collected only for participants with stroke such as: time post-stroke, stroke type, location, and severity using the National Institutes of Health Stroke Scale (maximum score 42, higher scores indicate greater severity) (44).

#### Cognitive function

Global cognitive function was assessed using the Montreal Cognitive Assessment (MoCA) (45), which assesses attention, memory, visuospatial abilities, language, abstraction, concentration and orientation domains of cognition. The maximum possible score for the MoCA is 30, where higher scores indicate better function and scores <26 suggesting mild cognitive impairment (45). While the MoCA is generally used as a screening tool for cognitive impairment, previous studies have also used the MoCA and similar screening tools such as the Mini-Mental State Examination (MMSE) to explore the association between global cognitive function and walking capacity among older adults (7–14). The MoCA has excellent sensitivity and specificity in its ability to detect mild cognitive impairment, excellent internal consistency and concurrent validity with the MMSE (45), and excellent inter-rater reliability in older adults (46). The MoCA also has high sensitivity and specificity for detecting cognitive impairment in a cardiovascular population (47), and is feasible for cognitive screening in individuals with stroke (48), with excellent internal consistency and high concurrent validity with the MMSE (49, 50).

#### Walking capacity

The 6 MWT (6) was used to assess functional capacity. Standardized guidelines were followed (51). Participants were instructed to walk as far as possible over 6 min around a 65-meter indoor oval track, and total distance was measured using a measuring wheel. In a subset of 8 participants, the 6 MWT was conducted along a 20-meter straight indoor hallway due to space limitations. Total distance walked, resting and peak blood pressure, heart rate, and rate of perceived exertion were recorded. Participants were permitted to use gait aids and to take rest breaks if needed. Assessors refrained from providing encouragement throughout the test.

In older adults, the 6 MWT has high test-retest reliability and convergent validity with maximal treadmill testing (52). The 6 MWT has also demonstrated discriminant validity in its ability to distinguish between adults 60–69 years and 80–89 years of age, and between low- and high-active individuals (52). In individuals with stroke, the 6 MWT has excellent test-retest reliability (53), and concurrent validity with the Functional Independence Measure locomotion and motor subscales (54), V˙O_2_peak (53, 55), and preferred and fast walking speed (56).

### Statistical analyses

Participants were included in the present analysis if they had data available on: (1) biological sex (2 levels: Male, Female); (2) walking capacity (6 MWT distance) and global cognitive function (MoCA); and (3) covariates of age, height and history of stroke (2 levels: Yes, No).

Participant demographics were described using means and standard deviations for normally distributed continuous variables, and medians and interquartile ranges for skewed data. Categorical variables were described using frequencies and percentages. All data was analyzed using StataIC Version 15 (StataCorp, College Station TX, USA) with an alpha set *a priori* at 0.05.

Multivariable regression analyses were performed to determine sex-based differences in the association between walking capacity (6 MWT distance, independent variable) and global cognitive function (MoCA score, dependent variable). We inspected variance inflation factors to ensure that multicollinearity was absent, and data were visually inspected for outliers and distribution. Assumptions for normality were tested using the Shapiro-Wilk test, while assumptions for homogeneity of the variances were tested using the Cook-Weisberg test. Residuals and leverage of the datapoints were examined for outliers. Any data points that influenced the beta-coefficients of the respective models by ≥10%, were deemed influential and thus removed (57).

Given our sample size of 59 participants, up to 6 variables were included in the regression models (58). We presented the unadjusted model (Model 1), then the model adjusted for age, history of stroke, and height (Model 2). Age and history of stroke were included due to their known associations with walking capacity (19, 23, 59–64) and global cognitive function (18, 65), and height was included to account for the known energy efficiency associated with walking with longer lower limb lengths (energy cost 2.6% lower for each 1 cm longer length) (66). To determine if there were sex-based differences in the association between global cognitive function and walking capacity, we included sex and an interaction term between sex*6 MWT distance in Models 3 and 4. The interaction term was subsequently removed, if deemed to be non-significant.

## Results

**Figure 1:**
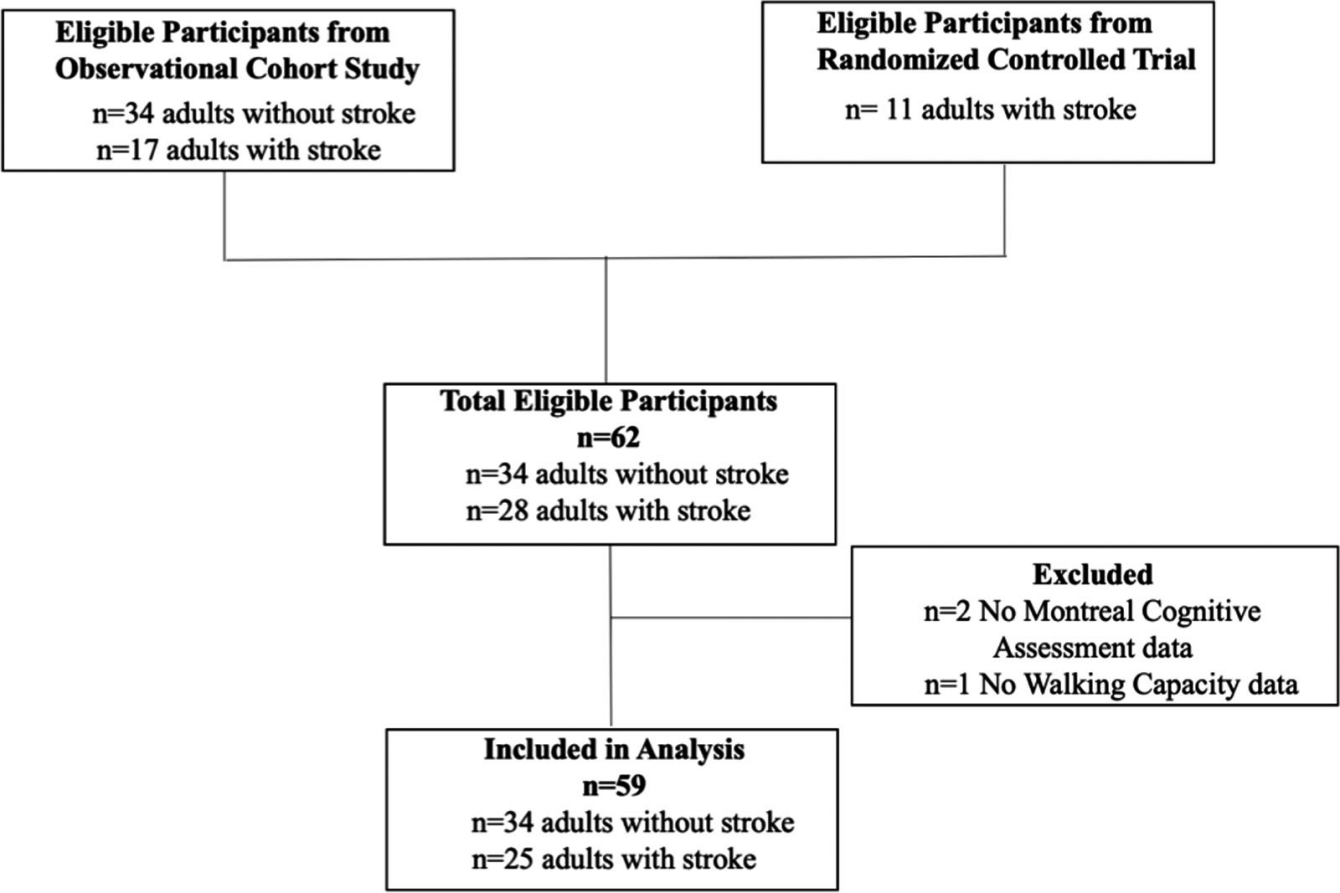
Participant flow through the study.

A flow chart of participants through the study is provided in Figure 1. Of 62 participants enrolled in both trials, the MoCA was not administered for two individuals with stroke due to aphasia and a language barrier, and 6 MWT was not performed for one individual with stroke due to significant knee pain. Therefore, 59 participants (23 females and 36 males) were included in the analyses. Participant characteristics are described in Table 1 for the entire sample, and disaggregated by sex.

**Table 1:** Participant demographics for full sample (*n* = 59) and disaggregated by sex.

	All (*N* = 59)	Males (*N* = 36)	Females (*N* = 23)	*p*-value for sex differences
Age (years), median (IQR), min-max	68.0 (10.0), 42–78	69.0 (12.0), 42–78	68.0 (6.0), 47–76	0.70
Montreal Cognitive Assessment score, median (IQR), min-max	25 (5.0), 13–30	24 (5.0), 13–30	26 (4.0), 17–29	0.08
6-Minute Walk Test distance (meters), mean (SD), min-max	473.1 ± 115.2, 203–750	474.8 ± 130.9, 203–750	470.4 ± 87.7, 253–601	0.89
Height (cm), mean ± SD, min-max	170.3 ± 8.8, 152–190	175.0 ± 7.5, 159–190	162.9 ± 4.7, 152–171	<0.01*
Gait aid *n* (%)				
None	54 (91.5)	33 (91.7)	21 (91.3)	0.93
Cane	4 (6.8)	3 (8.3)	1 (4.3)	0.55
Rollator	1 (1.7)	0 (0)	1 (4.3)	0.21
Completed Highschool, *n* (%)	54 (91.5)	33 (91.7)	21 (91.3)	0.93
Resting Systolic Blood Pressure (mmHg), mean ± SD, min-max	130 ± 13.9, 101–159	133.8 ± 2.3, 104–159	123.6 ± 2.5, 101–145	<0.01*
Resting Diastolic Blood Pressure	73.9 ± 9.8, 53–100	77.8 ± 9.4, 60–100	67.3 ± 6.4, 53–79	<0.01*
Resting Heart Rate (bpm), median (IQR), min-max	63 (16), 45–92	63 (15), 45–92	63.5 (15), 48–79	0.81

IQR, Interquartile range; SD, Standard Deviation; mmHg, millimetre of mercury; bpm, beats per minute.

*p < 0.05.

For the 25 individuals with stroke, median time post-stroke was 3.3 years (IQR: 2.3) and were of mild to moderate severity (NIH-SS = 1.9 ± 1.2). Fifteen individuals (60%) experienced ischemic stroke, 5 (20%) experienced hemorrhagic stroke, and 5 (20%) were of unknown origin. Participants without stroke were older than those with stroke (70 (IQR: 6.0) vs. 62 (IQR: 14.0)). There were no sex differences in MoCA scores and 6 MWT distances among the individuals with stroke.

The whole sample represented a broad range of global cognitive impairment and walking capacities. MoCA scores ranged from 13 (severe cognitive impairment) to 30 (maximum score, no evidence of impairment) (45). The median MoCA scores for males was 24 (IQR: 5.0) and 26 (IQR: 4.0) for females, respectively. Distances walked on the 6 MWT were similarly broad, ranging from 203 to 750 meters, and representing 39–144% of age-matched reference values for males [mean (SD): 474.8 ± 130.9 meters], and 56–133% for females [mean (SD): 470.4 ± 87.7 meters] (67).

**Figure 2:**
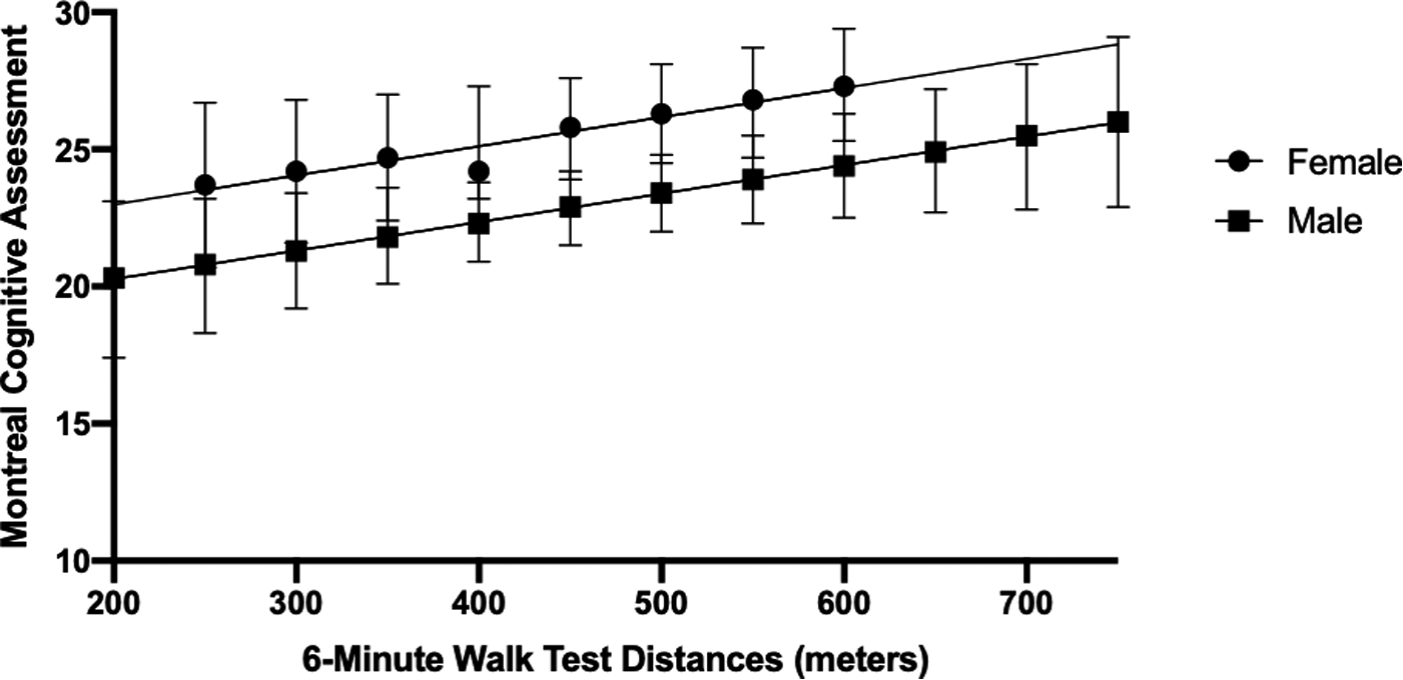
Marginsplot of sex differences in the association between global cognitive function (Montreal Cognitive Assessment scores) and Walking Capacity (6-Minute Walk Test distance) when adjusting for age, height and history of stroke. Main effect of sex (*p* < 0.05). Bars depict the 95% Confidence Intervals.

In the regression analysis, one outlier was removed due to its substantial influence (≥10%) on the beta-coefficient (*n* = 58 included in the final regression analysis). In the unadjusted model, 6 MWT distance was associated with global cognitive function, explaining 17% of the variance in MoCA scores (*R*^2 ^= 0.17; *F*(1,56) = 11.44; *p* < 0.01) (Table 2; Model 1). In the model adjusted for age, history of stroke, and height, walking capacity was associated with global cognitive function (*R*^2^ = 0.20; *R*^2^ Change = 0.026; *F*(4,53) = 3.23; *p* = 0.02) (Table 2; Model 2). In Model 3, the interaction between sex and 6 MWT was deemed not significant and removed from the model. Sex alone was added to the final model, where a main effect of sex was observed (Model 4, *R*^2 ^= 0.26; *R*^2^ Change = 0.068; *F*(5,21) = 3.72; *p* = 0.03). Figure 2 depicts the observed main effect of sex in the relationship between MoCA scores and 6 MWT distance when adjusting for age, height and history of stroke, whereby the males MoCA scores were 2.9 ± 1.3 less than the mean MoCA scores for females.

**Table 2:** Univariate and multivariable regression analyses examining the relationship between global cognitive function (Montreal Cognitive Assessment, dependent variable) and walking capacity (6-Minute Walk Test distance, independent variable) with models unadjusted (Model 1) and adjusted (Model 2), and sex (Model 3 for interaction, Model 4 for main effect).

Variables	*R* ^2^	*R*^2^ Change	Unstandardized ß (SE)	95% CI	*p*-value
*Model 1: F (1,56) = 11.44*	0.170				**<0**.01*
6 MWT			0.01 (0.004)	0.005, 0.02	**<0**.01*
*Model 2: F (4,53) = 3.23*	0.196	0.026			**0**.02*
6 MWT			0.01 (0.005)	0.002, 0.02	**0**.02*
Age			−0.04 (0.07)	−0.18, 0.10	0.57
Stroke			−1.42 (1.1)	−3.68, 0.85	0.22
Height			−0.01 (0.06)	−0.12, 0.10	0.83
*Model 3: F (6, 51) = 3.31*	0.280	0.02			**<0**.01*
6 MWT			0.02 (0.008)	0.001, 0.03	**0**.04*
Age			−0.02 (0.07)	−0.16, 0.12	0.77
Stroke			−0.41 (1.2)	−2.75, 1.84	0.73
Height			0.12 (0.08)	−0.04, 0.27	0.13
Sex (Male)			1.63 (4.4)	−7.20, 10.46	0.71
Sex (Male)*6 MWT			−0.01 (0.009)	−0.03, 0.009	0.29
*Model 4: F (5,21) = 3.72*	0.264	0.068			**<0**.01*
6 MWT			0.01 (0.005)	0.0009,0.02	**0**.03*
Age			−0.03 (0.07)	−0.16, 0.11	0.70
Stroke			−0.52 (1.1)	−2.86, 1.81	0.66
Height			0.10 (0.07)	−0.05, 0.26	0.17
Sex (Male)			−2.90 (1.3)	−5.56, −0.23	**0.03** *

6 MWT, 6-Minute Walk Test; SE, Standard Error; Stroke, History of Stroke.

Bolded values and * indicate *p* < 0.05.

## Discussion

This study was the first to perform a sex-based analysis in the relationship between global cognitive function and walking capacity. The positive relationship between global cognitive function and walking capacity observed in the current study corroborate previous reports in older adults with less impairment in cognitive and physical function (12–14) and in populations with chronic conditions (6–11, 15–17). This body of evidence reinforces the importance of promoting exercise and physical activity behaviours to maintain cognitive and physical function among male and female older adults of all abilities. Indeed, exercise, in particular aerobic exercise and dual-task activities (cognitive tasks performed simultaneously with exercise), have the potential to improve physical fitness (68), and to induce structural and functional changes in the brain conducive for better cognitive functioning (69, 70). Older adults possessing high walking capacity have shown to have preserved grey matter (15) and reduced beta amyloid plaque accumulation (71) and are thus at lower risk for Alzheimer's disease (72).

While we did not observe an interaction between distance walked on the 6 MWT and sex in the association with cognitive function, there was a main effect of sex whereby males presented with lower MoCA scores than females across all distances of 6 MWT. Of note, the between-sex difference of 2.9 points in MoCA scores is clinically meaningful in older adult (73) and stroke (74) populations. Previous cohort studies have consistently reported that higher levels of physical activity participation are associated with reduced cognitive decline in older males (75). Thus, physical activity and exercise-based interventions may also be important for global cognitive function, particularly in older male adults.

The mechanisms underlying the apparent preservation of cognitive function in aging females relative to males are not entirely known, although neuroimaging studies may provide some insight. Older females present with greater gyrification (76) suggestive of attenuated brain atrophy, whereas older males have reduced white and grey matter parenchymal volume (77). Sex-specific morphological changes in the brain are consistent with observed functional differences as well, whereby females outperform males in areas of language, executive function, memory, attention and global cognition tasks from the third decade until the eighth decade of life (78–80).

Although beyond the scope of our current analysis, we acknowledge that hormonal influences may also be a contributing factor to the observed findings (81). Sex-based differences in cognitive function favouring younger adult females are also prominent in later adulthood in females with a history of estrogen supplementation or therapy use, suggesting a neuroprotective effect of estrogen even in older females (82–86). Estrogen aids in protecting against apoptosis and inflammatory processes such as beta amyloid accumulation, while also stimulating the synthesis of neurotransmitters, such as acetylcholine (87). Additionally, lower levels of circulating brain-derived neurotrophic factor, common in males (28, 29, 88), have been shown to be associated with poor cognitive function (89). Taken together, it may be that physiological and molecular mechanisms such as attenuated brain atrophy, the neuroprotective effect of estrogen in females with a history of estrogen supplementation or therapy, and higher levels of circulating neurotrophins critical to brain function may help explain the greater MoCA scores observed among older females across all levels of walking function in our adjusted model analyses.

### Limitations

We acknowledge there are limitations to this study. For the current analysis, we prioritized the key variables shown to be most influential on the relationship between walking capacity and global cognitive function. Future studies may explore additional variables that may influence the relationship between sex, walking capacity, and global cognitive function such as estrogen supplementation or estrogen replacement history (83–86). Due to the exploratory nature of this study, we also opted to use a broad assessment of global cognitive function using the MoCA rather than domain-specific measures. Males are known to achieve greater outcomes on tasks of visuospatial abilities, while females outperform males in areas of language, executive function, memory, as well as global cognitive function (78–80). Future research may expand on these findings by examining sex-based differences in the associations between specific cognitive domains with walking capacity in an aging population.

## Conclusion

Our findings confirm the positive relationship between cognitive and physical function in older adults. Notably, we also observed superior performance in global cognition among females that was consistent across a broad spectrum of walking capacity.

## Data Availability

The raw data supporting the conclusions of this article will be made available by the authors, without undue reservation.
